# Prediction of *Pseudomonas* spp. Population in Food Products and Culture Media Using Machine Learning-Based Regression Methods

**DOI:** 10.3390/life13071430

**Published:** 2023-06-22

**Authors:** Fatih Tarlak, Özgün Yücel

**Affiliations:** 1 Department of Nutrition and Dietetics, Istanbul Gedik University, Kartal, Istanbul 34876, Turkey; 2 Department of Chemical Engineering, Gebze Technical University, Gebze, Kocaeli 41400, Turkey; yozgun@gtu.edu.tr

**Keywords:** predictive microbiology, machine learning approach, *Pseudomonas* spp.

## Abstract

Machine learning approaches are alternative modelling techniques to traditional modelling equations used in predictive food microbiology and utilise algorithms to analyse large datasets that contain information about microbial growth or survival in various food matrices. These approaches leverage the power of algorithms to extract insights from the data and make predictions regarding the behaviour of microorganisms in different food environments. The objective of this study was to apply various machine learning-based regression methods, including support vector regression (SVR), Gaussian process regression (GPR), decision tree regression (DTR), and random forest regression (RFR), to estimate bacterial populations. In order to achieve this, a total of 5618 data points for *Pseudomonas* spp. present in food products (beef, pork, and poultry) and culture media were gathered from the ComBase database. The machine learning algorithms were applied to predict the growth or survival behaviour of *Pseudomonas* spp. in food products and culture media by considering predictor variables such as temperature, salt concentration, water activity, and acidity. The suitability of the algorithms was assessed using statistical measures such as coefficient of determination (R^2^), root mean square error (RMSE), bias factor (Bf), and accuracy (A_f_). Each of the regression algorithms showed appropriate estimation capabilities with R^2^ ranging from 0.886 to 0.913, RMSE from 0.724 to 0.899, B_f_ from 1.012 to 1.020, and A_f_ from 1.086 to 1.101 for each food product and culture medium. Since the predictive capability of RFR was the best among the algorithms, externally collected data from the literature were used for RFR. The external validation process showed statistical indices of B_f_ ranging from 0.951 to 1.040 and A_f_ ranging from 1.091 to 1.130, indicating that RFR can be used for predicting the survival and growth of microorganisms in food products. Therefore, machine learning approaches can be considered as an alternative to conventional modelling methods in predictive microbiology. However, it is important to highlight that the prediction power of the machine learning regression method directly depends on the dataset size, and it requires a large dataset to be employed for modelling. Therefore, the modelling work of this study can only be used for the prediction of *Pseudomonas* spp. in specific food products (beef, pork, and poultry) and culture medium with certain conditions where a large dataset is available.

## 1. Introduction

Predictive food microbiology integrates traditional knowledge of food microbiology with mathematics and statistics to develop statistical models that predict microbial behaviour in the food environment [1]. Although predictive models have been used for over a century, their development has greatly accelerated in the 21st century with the aid of computer technology [2]. These models are used to determine the conditions that can reduce or delay the harmful effects of microbial contamination of food. Traditional predictive food microbiology relies on primary and secondary models to simulate how microorganisms behave over time and in different environmental conditions [3]. Primary models, such as the modified Gompertz, logistic, Baranyi, and Huang models, are commonly utilised to describe microorganism behaviour under consistent environmental conditions. Secondary models, on the other hand, take into account the impact of environmental factors and food characteristics on the parameters of the primary model [4].

The prevalent and traditional modelling technique in predictive microbiology is the two-step modelling approach, which involves fitting the primary and secondary models sequentially. Initially, the primary model is fitted to growth data points, and then the resulting growth kinetic parameters are integrated into the secondary model, considering environmental factors such as temperature [5]. Nevertheless, the two-step modelling approach has its limitations. One significant drawback is the potential accumulation and propagation of errors resulting from the repeated sequential nonlinear regression process [4]. This leads to a notable level of uncertainty in the parameters of the secondary model, particularly when there is a scarcity of microbial data or significant biological variability. Additionally, accurately determining the duration of the lag phase becomes challenging in cases where there are inadequate growth data points or microorganisms exhibit short lag times. Consequently, these challenges can result in imprecise estimations. Moreover, the current approach overlooks poor estimations from the primary model during secondary modelling. The lack of consideration for the fit of individual growth curves means that all parameters estimated from observed values are treated equally in the second step, potentially leading to inaccuracies in the final estimates [6,7].

Machine learning is a subfield of artificial intelligence (AI) that focuses on the development of algorithms and models that enable computers to learn and make predictions or decisions without being explicitly programmed. It involves the use of statistical techniques and computational algorithms to analyse and interpret patterns in large datasets. Machine learning algorithms are designed to learn from data, identify patterns, and make accurate predictions or decisions based on the patterns they discover. These algorithms are typically trained using labelled data, where the input data is paired with corresponding desired output or target values [8]. During the training process, the algorithm adjusts its internal parameters to minimise the difference between its predicted outputs and the true target values [9,10].

The use of machine learning algorithms in food safety and modelling has gained popularity due to the collective possibilities of rapidly capturing large amounts of digital data, an increase in affordable computing power and data storage, and a global system of interconnected computer networks. Several published works have used machine learning applications in food safety and modelling. Golden et al. [9] employed various machine learning algorithms, including support vector regression, extremely randomised trees regression, and Gaussian process regression, to estimate the population growth of *Escherichia coli* O157. In a study conducted by Hiura et al. [10], the authors utilised the eXtreme gradient boosting tree, a machine learning algorithm, to make predictions about the bacterial population behaviour of *Listeria monocytogenes* in five different food categories, namely beef, culture medium, pork, seafood, and vegetables. In a different study by Tarlak and Yücel [11], a prediction tool was developed to characterise the behaviour of *Listeria monocytogenes* in milk. The authors employed both traditional models, such as the re-parametrised Gompertz, Baranyi, and Huang models, as well as a machine learning-based regression model. Yücel and Tarlak [12] developed a prediction tool to describe the behaviours of *Listeria monocytogenes*, *Escherichia coli*, and *Pseudomonas* spp., specifically in beef. Collectively, all these studies highlighted the potential of machine learning models in predicting the behaviour of bacterial populations.

This study employed a data mining approach to estimate the behaviour of bacterial populations in different food products and culture media by gathering previously published data. The study focused on *Pseudomonas* spp., one of the most common microorganisms that directly cause food spoilage [13], and used machine learning-based regression methods, such as support vector regression, Gaussian process regression, decision tree regression, and random forest regression, to model the change in the *Pseudomonas* spp. population over time. The best-performing regression method was externally validated using the bias factor and accuracy factor for predicting bacterial *Pseudomonas* spp. counts and an interface was developed to be used for the estimation of bacterial counts of *Pseudomonas* spp. This work introduces several novel aspects, including (i) a comprehensive comparison of machine learning regression methods for predicting the survival and growth manner of the *Pseudomonas* spp. population over time, (ii) the development of a user-friendly interface that enables the prediction of bacterial count for *Pseudomonas* spp. based on various parameters, including time, temperature, NaCl concentration, water activity, CO_2_ concentration, vacuum conditions, and food category. This interface facilitates the understanding of microorganism survival and growth patterns, offering a practical tool for describing *Pseudomonas* spp. behaviour.

## 2. Materials and Methods

**Figure 1:**
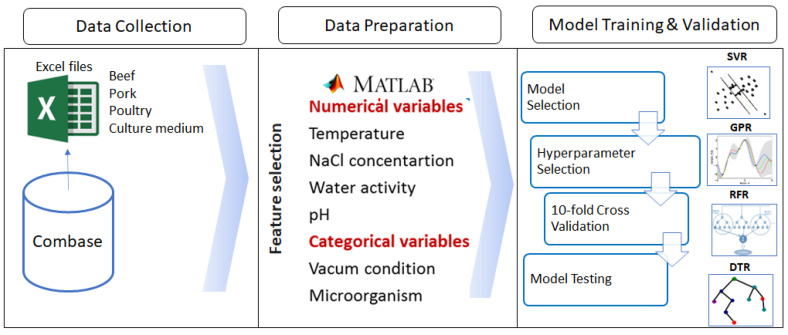
The flow chart outlining the main steps followed in the present study.

The work was conducted in three separate main steps: (i) the bacterial data points of *Pseudomonas* spp. in various food products (beef, pork, and poultry) and culture media were gathered from the ComBase database (www.combase.cc, accessed on 1 June 2021), (ii) data processing (data ingestion, standardisation, and featurisation) was performed in Matlab 8.3.0.532 (R2014a) software (MathWorks Inc., Natick, MA, USA), and (iii) various machine learning-based regression methods including support vector regression, Gaussian process regression, random forest regression, and decision tree regression were employed for estimation of the *Pseudomonas* spp. population using Matlab 8.3.0.532 (R2014a) software. The evaluation of machine learning-based regression methods involved assessing their estimation power using several metrics, including the coefficient of determination, root mean square error, bias factor, and accuracy factor. Figure 1 presents a flow chart illustrating the main steps followed in the current study. The subsequent subsections provide detailed descriptions of each stage in this work.

### 2.1. Data Collection

The ComBase database (www.combase.cc, accessed on 1 June 2021) provides almost 60,000 systematically formatted and quantified microbial records gathered from numerous research institutions and papers. In this database, microbial responses are available with their information, including “record ID”, “organism”, “food category”, “food name”, “temperature”, “pH”, “water activity”, “conditions”, “time”, and “viable cell counts”, which enables us to separately categorise and sort out experimental sets of microorganisms. So that the growth or survival manner of *Pseudomonas* spp. could be modelled using machine learning-based regression methods, all data points of *Pseudomonas* spp. available in the ComBase database were collected and employed in this work. Three kinds of food products, including beef, pork, and poultry, and culture media were considered because they have an adequate number of data points for *Pseudomonas* spp. For modelling based on the machine learning approach, all data points were stored with their information of record ID, temperature, NaCl concentration, water activity, pH, CO_2_ concentration, vacuum condition, time, microbial population, food category, and food name. All available data points in the Combase database for beef, pork, poultry, and culture media were collected, but the information regarding temperature, pH, and water activity was not available for some datasets. The datasets lacking at least one value regarding temperature, pH, and water activity were not considered in developing the model. A total of 282 data points for beef, 595 data points for pork, 426 data points for poultry, and 4315 data points for culture media collected from the ComBase database were employed for model development and assessment. Timeline and reference information regarding all used bacterial data points of *Pseudomonas* spp. can be found in the ComBase database in detail, and their corresponding record ID codes are given in Supplementary Data S1.

### 2.2. Data Pre-Processing

The bacterial count of *Pseudomonas* spp. in the unit of log CFU was defined as the main objective function considering the entire dataset categorised into numerical and categorical values for each record ID. The parameters “time”, “temperature”, “NaCl concentration”, “water activity”, and “CO_2_ concentration” are numerical data. The microbial counts (log CFU/g) at 0 h were determined as the initial count of *Pseudomonas* spp. for each record ID. To separate initial counts from others, data belonging to a time of 0 (h) were coded as 0, and other data were coded as 1. Through this process, the information on the initial count of *Pseudomonas* spp. was also converted to numerical data. The parameters, vacuum condition (yes/no), food category (beef, pork, poultry, and culture medium), and food name (minced beef, pork, raw meat lombo, turkey, brain heart infusion broth “BHIB”, and several kinds of tryptic soy broth “TSB”), are categorical data and were kept as is. These variables were not transformed into numerical values, and they were directly used for predictions to avoid the possibility that the machine learning algorithms can create bias in the encoded variables by assuming that higher numbers are more important. The pre-processing steps were performed using Matlab 8.3.0.532 (R2014a) software (MathWorks Inc., Natick, MA, USA).

### 2.3. Modelling

The predictive capability of machine learning models varies depending on the data bias and variance. Support vector machine (SVM) is a popular non-parametric technique for classification and regression that transforms data into hyperspace to find linear or nonlinear relationships between predictors and responses. SVM relies on kernel functions to define the feature space where data are regressed. The radial basis function kernel is commonly used for support vector regression. However, its effectiveness decreases with noise in the dataset [12,14].

Gaussian process regression (GPR) is a flexible, fully probabilistic, and non-parametric Bayesian approach. It is based on the concept of an infinite-dimensional generation of normal distributions with multivariate Gaussian distribution. GPR constructs objective functions based on the distance measure between the estimated output probability density function (PDF) for a given dataset. GPR maintains high certainty in unsampled locations far from the training data. However, it takes into account the entire training data each time it makes a prediction, resulting in an expensive computational effort [11,15].

Decision tree regression (DTR) is a non-parametric and interpretable algorithm frequently used for regression or classification problems [16]. It gives not only predictions but also inferences about the data. Data pre-processing is simplified when using DTR, as it eliminates the need for data scaling. Additionally, DTR can handle categorical features without requiring numerical encoding. To mitigate bias and variance issues, ensemble methods are frequently employed. These methods involve combining multiple decision trees to achieve enhanced predictive performance. However, DTR is inadequate for regression and is better suited for classification [17,18].

Random forest regression (RFR) fits a large number of classification trees to a dataset and combines their predictions to produce a final predictive model [12,19]. RFR is effective in finding nonlinear relationships in the training data and generalises well to new data. It is not sensitive to outliers, and the use of the entire forest rather than an individual tree helps avoid overfitting the model to the training dataset while discovering the relationships between the predictors and response. Boosting algorithms are commonly employed for RFR [20,21].

### 2.4. Assessment of the Quality of Fit

To compare the performance of the models, several metrics were utilised, including the coefficient of determination (R^2^), root mean square error (RMSE), bias factor (B_f_), and accuracy factor (A_f_). These metrics were calculated using the Equations (1)–(4), respectively [4]:

(1) $$R^{2}=1-\left[\frac{\sum_{i=1}^{n}{\left(y_{obs}-y_{pre}\right)}^{2}}{\sum_{i=1}^{n}{\left(y_{obs}-\bar{y_{obs}}\right)}^{2}}\right]$$

(2) $$\mathrm{RMSE}=\sqrt{\frac{\sum_{i=1}^{n}{\left(y_{obs}-y_{pre}\right)}^{2}}{n}}$$

(3) $$B_{f}=10^{\frac{\sum_{i=1}^{n}log\left(y_{pre}/y_{obs}\right)\;}{n}}$$

(4) $$A_{f}=10^{\frac{\sum_{i=1}^{n}\left|log\left(y_{pre}/y_{obs}\right)\right|}{n}}\;$$

where *y_obs_* is the experimental bacterial population, *y_pre_* is the predicted value, $\bar{y_{obs}}$ is the average of the population count, and *n* is the observation number.

**Figure 2:**
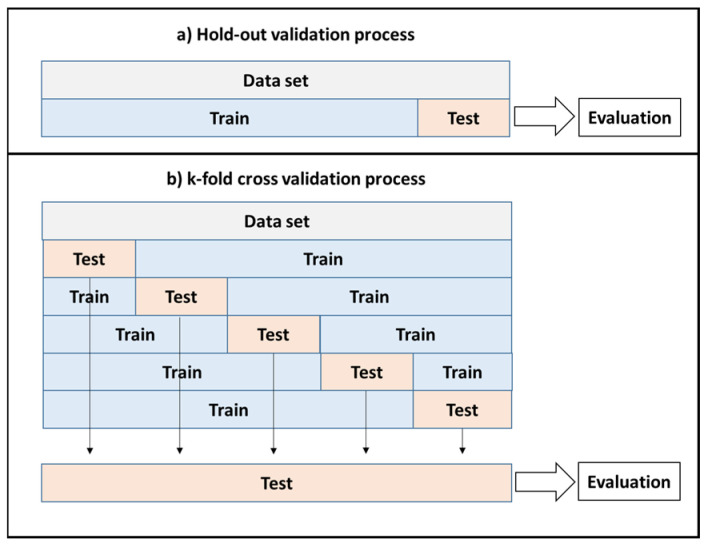
The schematic illustration of validation methods of (**a**) hold-out validation and (**b**) k-fold validation.

The two most commonly used validation methods in machine learning are hold-out and k-fold [22]. Hold-out validation involves dividing the dataset into two sets: training and test. The model is then trained on the training set and evaluated on the test set to assess its performance. In k-fold cross-validation, the dataset is divided into k-equal partitions. In each iteration, one partition is used for testing, and the remaining partitions are used for training. The results from all iterations are combined to provide predictions for the entire dataset. Cross-validation provides an unbiased evaluation, whereas hold-out validation can introduce bias because the splitting process is random. The validation methods are illustrated in Figure 2. A 10-fold cross-validation method was employed in this study.

## 3. Results and Discussion

**Figure 3:**
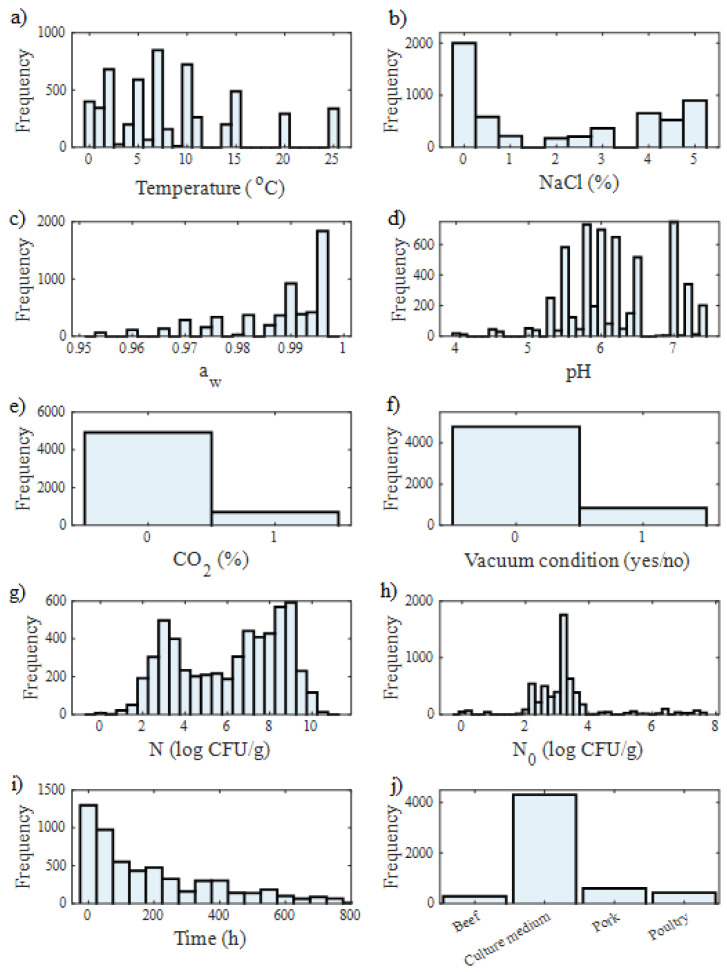
Histograms depicting the variables are shown for (**a**) temperature (°C), (**b**) NaCl concentration (%), (**c**) water activity, (**d**) pH, (**e**) CO_2_ concentration (%), (**f**) vacuum condition (yes/no), (**g**) initial microbial count (log CFU/g), (**h**) microorganism population (log CFU/g), (**i**) time, (**h**,**j**) food category.

The growth and survival data points of *Pseudomonas* spp. in various food products (beef, pork, and poultry) and culture media collected from the ComBase database were stored with the following information: record ID, temperature (°C), NaCl concentration (%), water activity, pH, CO_2_ concentration (%), vacuum condition (yes/no), initial microbial population (yes/no), time (h), and food category. The data frequency of the collected data categorised into each feature is shown in Figure 3. A total of 282, 4315, 595, and 426 growth and survival data points were employed for beef, culture medium, pork, and poultry, respectively. Furthermore, Table 1 presents the minimum and maximum ranges of each main predictor variable which directly influences the behaviour of *Pseudomonas* spp. The corresponding standard deviations (σ) are also provided.

The maximum specific growth rate (μ_max_), which is one of the most important growth kinetic parameters, can be modelled with respect to environmental factors such as temperature, NaCl concentration, water activity, and pH. Among these factors, temperature plays a key role in affecting microbial growth behaviour in food [5]. Temperature variables ranged from 2 to 11 °C for beef, 0 to 25 °C for culture medium, 0.1 to 10.4 °C for pork, and 1 to 7 °C for poultry, which means 5618 collected growth data points were in the range of 0 to 25 °C which are real temperatures to which food products are subject to in storage, delivery, and retail marketing processes. NaCl concentration (%) ranged from 0 to 5% for the culture medium, while there was no NaCl for beef, pork, and poultry; 3624 NaCl concentration data were collected for the culture medium. This information was used for the prediction of *Pseudomonas* spp. in culture medium and pork, which means 64% of collected datasets of *Pseudomonas* spp. growth data contributed as a predictor variable in total. The water activity of a food product is the ratio between the vapour pressure of the food itself when in a completely undisturbed balance with the surrounding air media and the vapour pressure of distilled water under identical conditions [23]. Most foods have a water activity above 0.95, which provides sufficient moisture to support the growth of microorganisms. In this work, water activity was in the range of 0.95 to 1 for each of the food products. Another important factor that directly affects the growth behaviour of microorganisms is pH. In this study, pH ranged from 5.82 to 5.9 for beef, 4.01 to 7.40 for culture medium, 5.30 to 6.00 for pork, and 6.00 to 6.20 for poultry.

**Figure 4:**
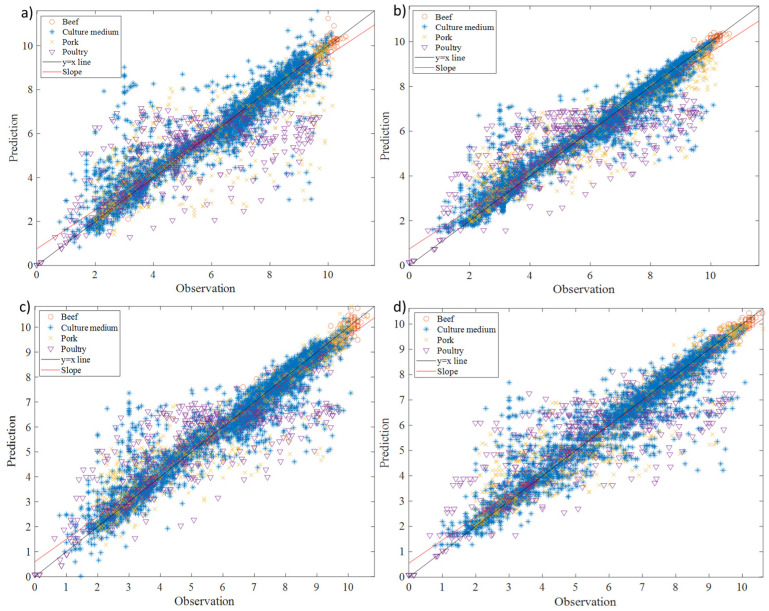
The observed and predicted *Pseudomonas* spp. in different food products and culture media using (**a**) support vector machine regression, (**b**) Gaussian process regression, (**c**) decision tree regression, and (**d**) random forest regression.

The predictive performance of different machine learning-based regression methods (support vector regression, Gaussian process regression, decision tree regression, and random forest regression) in estimating *Pseudomonas* spp. behaviour was assessed by evaluating their statistical indices (R^2^, RMSE, B_f_, and A_f_). The correlations between the observed and predicted values are illustrated in Figure 4, showcasing the results for support vector machine regression, Gaussian process regression, decision tree regression, and random forest regression, respectively.

The range of R^2^ values obtained from the machine learning-based regression methods for all food products (beef, pork, and poultry) and culture media was 0.866 to 0.913, while the corresponding RMSE values ranged from 0.724 to 0.899 (Table 2). In a study by Hiura et al. [10], a machine learning algorithm was employed to predict the behaviour of *Listeria monocytogenes* in various food products such as beef, culture medium, and pork. The reported R^2^ and RMSE values were up to 0.80 and at least 0.96, respectively. Comparatively, the machine learning-based regression methods utilised in our study (support vector regression, Gaussian process regression, decision tree regression, and random forest regression) demonstrated notably superior prediction capabilities than the method employed by Hiura et al. [10] for predicting *Listeria monocytogenes* behaviour. Moreover, despite skipping the traditional secondary modelling step for determining the effects of environmental factors and/or food matrices on model parameters, the support vector regression, Gaussian process regression, decision tree regression, and random forest regression used in this study displayed excellent prediction capability, with 1.012 < B_f_ < 1.017 and 1.086 < A_f_ < 1.101. Among these methods, the decision tree regression had B_f_ and A_f_ values of 1.012 and 1.086, respectively (Table 2), where a B_f_ of 1 indicates no structural deviation of the model. The B_f_ value of 1.012 indicated that the model overestimated by 1.2%, while the A_f_ factor of 1.086 showed that, on average, the predicted value differed from the observed value by 8.6% (either smaller or larger). These values were slightly better than those obtained for support vector regression, Gaussian process regression, and decision tree regression. As a result, the random forest regression was selected as the optimal regression procedure and further analysed for its prediction capability for each food product.

**Figure 5:**
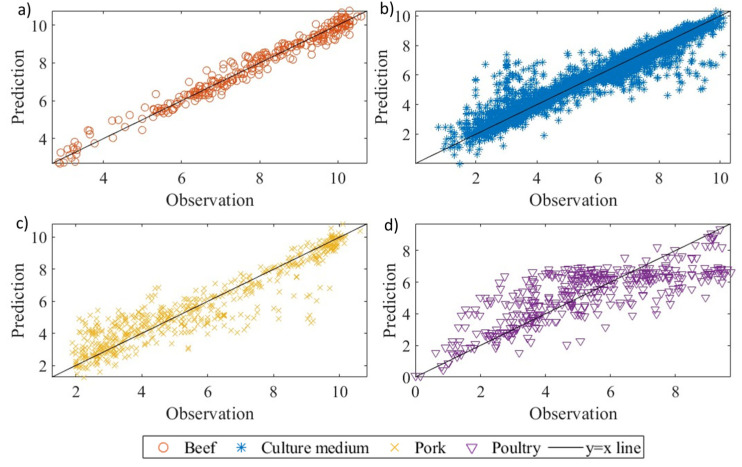
The observed and predicted *Pseudomonas* spp. in (**a**) beef, (**b**) culture media, (**c**) pork, and (**d**) poultry using random forest regression.

The prediction capability of random forest regression was also evaluated separately by food category. Figure 5 shows that the random forest regression yielded good prediction performance for each of the food categories (beef, pork, and poultry) and culture media. However, the prediction power of decision tree regression was the best for modelling *Pseudomonas* spp. in beef, followed by culture medium, pork, and poultry. Furthermore, Table 3 provides a summary comparing the prediction capability of the decision tree regression used in this study with the machine learning algorithm employed by Hiura et al. [10] for predicting the population of *Listeria monocytogenes*. Random forest regression used in this study provides considerably better goodness-of-fit indices of 0.861 < R^2^ < 0.973, 0.326 < RMSE < 0.968, 1.006 < B_f_ < 1.052, and 1.086 < A_f_ < 1.408 for beef, culture medium, and pork than Hiura et al. [10].

In general, it is always better to use the k-fold technique instead of hold-out. K-fold gives more stable and trustworthy prediction results since training and testing processes are performed on several different parts of the dataset. On the other hand, the hold-out method involves splitting a dataset into 20–30% test data with the rest as training data. These numbers can vary—a larger percentage of test data will make the model more prone to errors as it has less training experience, while a smaller percentage of test data may give the model an unwanted bias towards the training data. This lack of training or bias can lead to underfitting/overfitting of the model [24]. In this study, the k-fold cross-validation method was used, and k was chosen as 10 to estimate the error in an unbiased way. Hiura et al. [10] used the hold-out method; therefore, the evaluation of the performance of the employed machine learning algorithm can vary with the splitting process. This shows that the prediction results and prediction capability evaluations in the current work are more reliable than Hiura et al. [10] reported. However, Hiura et al. [10] presented a new pioneering perspective to estimate microorganism behaviour using a machine learning approach.

**Figure 6:**
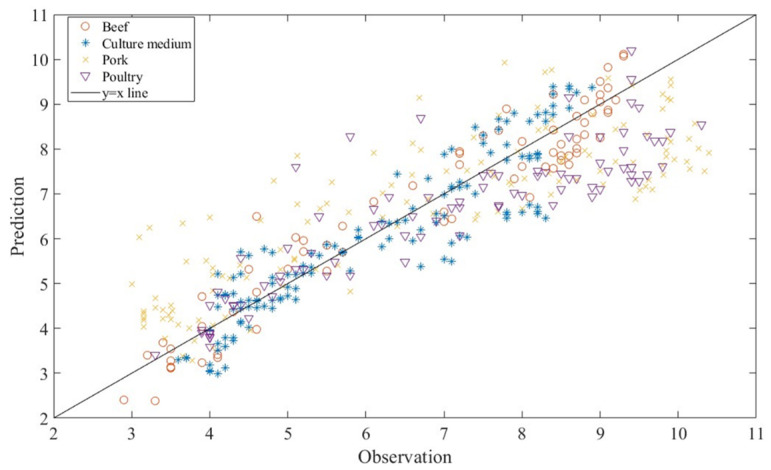
The observed and predicted *Pseudomonas* spp. using random forest regression for external validation.

For reliable utilisation of the developed models, it is crucial to perform external validation through independent experiments. Therefore, the data obtained from the independent experiments on beef [25,26], chicken [27,28], pork [25,27,29,30], and culture medium [31] were compared with the predicted number of *Pseudomonas* spp. with the random forest regression used by considering the B_f_ and A_f_ values (Figure 6). The B_f_ and A_f_ values were found to be 1.028 and 1.236, respectively. A B_f_ factor of 1 indicates no structural deviation of the model. The B_f_ factor of 1.028 indicated that the model overestimates by 2.8%, whereas the A_f_ factor of 1.236 showed that, on average, the predicted value was 23.6% different (either smaller or larger) from the observed value. These results revealed that the random forest regression could be safely used because the error rates are relatively small. 

Maximum specific growth rate (µ_max_) and lag phase duration (λ) are the most critical parameters to describe the growth behaviour of microorganisms in food [32]. Both these parameters could not be directly determined, although total *Pseudomonas* spp. can be predicted using the developed model based on machine learning regression. Therefore, this may be considered the first limitation of this methodology when compared with traditional modelling methods in the predictive microbiology area. Despite the limitations of machine learning regression models in directly predicting the λ and µ_max_ of microorganisms on the food products, these parameters can still be calculated using the graphical approach. By plotting the population size against time and visually examining the curve’s shape and slope, λ can be estimated by identifying the point where the growth curve deviates from the baseline and starts to increase exponentially. To calculate µ_max_, the growth rates from the steepest part of the growth curve can be averaged or the median taken, representing the maximum rate of growth under the specific experimental conditions. The graphical approach provides a valuable method for estimating these critical parameters in the study of microbial growth behaviour [28]. 

As a second limitation, the prediction power of the machine learning regression method directly depends on dataset size. If there are not enough data, the machine learning method may not be used for the prediction of microorganism behaviour, meaning it requires a large dataset to be employed for modelling. When modelling microbial growth, utilising a larger dataset yields improved estimations and reduces uncertainty in model parameters. However, incorporating substantial amounts of microbial data into traditional primary and secondary models poses challenges, resulting in high uncertainty in model parameters and estimations due to limited degrees of freedom caused by a scarcity of microbial data or the significant biological variation observed in certain cases. On the other hand, employing a machine learning approach is well-suited for handling large datasets. Initially perceived as a limitation, this aspect can actually be considered an advantage when striving for accurate predictions. 

Additionally, this modelling work can only be used for the prediction of *Pseudomonas* spp. in specific food products (beef, pork, and poultry) and culture medium with certain conditions. However, this situation is also valid for all the modelling works carried out with traditional modelling methods in the predictive microbiology area. On the other hand, the machine learning approach enables simultaneous modelling of microbial survival and growth behaviour, which can be considered the most important advantage, as it is impossible to perform using traditional modelling approaches (primary, secondary, and tertiary models) in the predictive microbiology area.

## 4. Conclusions

In this study, different machine learning-based regression methods (support vector regression, Gaussian process regression, decision tree regression, and random forest regression) were used to estimate the count of *Pseudomonas* spp. in various food products (beef, pork, and poultry) and culture media. The performance of all regression algorithms was satisfactory, but the random forest regression showed the best estimation power. To further test its prediction capability, the algorithm was validated using external data from the literature. The statistical indices obtained for all food products combined were 0.951 < B_f_ < 1.040 and 1.091 < A_f_ < 1.130. Despite the random forest regression displaying favourable prediction capabilities for each food product individually, the most accurate estimations were observed specifically for the beef category. The results suggest that random forest regression can be a reliable alternative for describing the survival and growth manner of microorganisms in food products and has the potential to be used as a simulation method by skipping the secondary model step in the conventional two-step modelling method used in predictive microbiology.

## Figures and Tables

**Table 1:** Comprehensive details regarding the experimental conditions.

Food Products	Temperature (°C)			NaCl Concentration (%)			Water Activity			pH		
	Min.	Max.	σ	Min.	Max.	σ	Min.	Max.	σ	Min.	Max.	σ
Beef	2.00	11.00	3.32	-	-	-	0.99	0.99	0.00	5.82	5.90	0.04
Culture medium	0.00	25.00	7.07	0.00	5.00	1.93	0.95	1.00	0.01	4.01	7.40	0.70
Pork	0.10	10.40	3.16	-	-	-	0.98	0.99	0.00	5.30	6.00	0.22
Poultry	1.00	7.00	2.95	-	-	-	0.99	0.99	0.00	6.00	6.20	0.10

**Table 2:** The fitting capabilities of various machine learning regression methods.

Regression Methods	R^2^	RMSE	B_f_	A_f_
Support vector regression	0.866	0.899	1.017	1.101
Gaussian process regression	0.910	0.738	1.020	1.095
Decision tree regression	0.910	0.737	1.012	1.096
Random forest regression	0.913	0.724	1.012	1.086

**Table 3:** Performance evaluation of random forest regression for various food products.

	Hiura et al. [10]			This Study		
	Beef	Culture Medium	Pork	Beef	Culture Medium	Pork
data points	2887	77	1497	282	4315	595
R^2^	0.75	0.74	0.80	0.973	0.938	0.861
RMSE	1.02	1.15	0.96	0.326	0.600	0.968
B_f_	0.98	0.99	0.91	1.006	1.019	1.052
A_f_	1.47	1.37	1.46	1.086	1.185	1.408

## Data Availability

When a reader expresses interest, data can be provided. This information can be shared here.

## Supplementary Materials

The following supporting information can be downloaded at: https://www.mdpi.com/article/10.3390/life13071430/s1.
